# Using a Mobile Diary App in the Treatment of Borderline Personality Disorder: Mixed Methods Feasibility Study

**DOI:** 10.2196/12852

**Published:** 2019-09-30

**Authors:** Stig Helweg-Joergensen, Thomas Schmidt, Mia Beck Lichtenstein, Susanne S Pedersen

**Affiliations:** 1 Department of Psychology University of Southern Denmark Odense Denmark; 2 Center for Telepsychiatry Mental Health Services in the Region of Southern Denmark Odense Denmark; 3 The Borderline DBT-Unit Mental Health Services in the Region of Southern Denmark Svendborg Denmark; 4 The Maersk Mc-Kinney Moller Institute University of Southern Denmark Odense Denmark; 5 Department of Clinical Research University of Southern Denmark Odense Denmark; 6 Department of Cardiology Odense University Hospital Odense Denmark

**Keywords:** borderline personality disorder, mHealth, implementation, focus groups, e-diary, mobile app

## Abstract

**Background:**

Borderline personality disorder (BPD) is a disorder characterized by difficulties with regulating emotions and impulsive behavior. Long-term monitoring of progress during BPD psychotherapy constitutes a challenge using paper and pencil registration. Hence, a mobile app assessing emotions and progress in treatment may be useful.

**Objective:**

The aim of this study was to examine the feasibility of using the mDiary app as an adjunct to dialectical behavior therapy (DBT) for the treatment of BPD.

**Methods:**

A total of 9 focus group interviews were conducted and analyzed according to the grounded theory approach. Furthermore, the usability of the mDiary app was examined using the System Usability Scale (SUS). The app was implemented in a standard DBT program as an adjunct to DBT. In total, 16 patients (age range 19-41 years) and 23 therapists (age range 25-64 years) from 5 Danish public outpatient psychiatric treatment facilities participated in the study.

**Results:**

Overall, patients were satisfied with the mDiary app, as it was “easy to use” and “always there.” Inside-out innovation, meaning new work tasks generated during implementation and communication of modifications needed in the app, was found to influence the perceived usability negatively among the interviewed therapists. The patients rated the usability as high (mean SUS score 81.2, SD 9.9), whereas therapists rated the mDiary app at an average level (mean 68.3, SD 14.3). Older age of the users correlated with lower usability ratings on the SUS score (Pearson *r*=−0.60).

**Conclusions:**

The mDiary app was considered as an acceptable and relevant way of registering DBT diary data for both patients and therapists generating increased long-term overview. Older users were overall more reluctant to accept this new technology in clinical practice. Time to align expectations among involved parties needs to be set aside when implementing this new approach to patient monitoring. Here, the focus should be on the realistic use of resources and expected impact on present clinical work.

## Introduction

Borderline personality disorder (BPD) is characterized by emotional instability across a number of domains: mood, interpersonal relationships, self-image, impulse, and behavioral control. Generally, these BPD manifestations are attributed to a lack of ability to regulate emotions. Norwegian female patients with a personality disorder have a 38-fold increased risk of death by suicide compared with the general population [1]. In Denmark, Sweden, and Finland, the mortality of patients, who at some point have been admitted to hospital because of a mental disorder, is shown to be 2 to 3 times higher than in the general population [2]. It is estimated that between 1% and 5% of the Scandinavian population and 1.5% of the population in the Western world meet the criteria for BPD [3-5]. Around 10% of BPD patients will die from suicide, with most of these deaths occurring before patients reach 40 years of age [6]. The prevalence of BPD in clinical populations is estimated to be around 28% (range 9.3%-46.3% of patients across studies) [6].

Dialectical behavior therapy (DBT) has shown good clinical efficacy and is regarded as 1 of the most well-researched evidence-based treatments for BPD [5,7-9]. The main focus of DBT treatment is the learning of a predefined set of behavioral skills that target lack of emotional, mental, interpersonal, and behavioral control [10]. In standard clinical practice, evaluation of a patient’s progress in learning DBT skills is left to the clinician’s subjective memory and the weekly evaluation of paper-based client diary cards [11]. Although it is possible to go back and review progress over time, this is very time consuming and outside the realistic use of resources when using weekly paper-based diary cards in psychotherapy. An advantage when comparing app-registration with paper diaries would seem to be the addition of a long-term overview of patients’ scores and a better overview of patient-acquired DBT skills [12].

Self-monitoring would logically reduce patient burden, increase compliance in registration, and generate new opportunities for long-term overview of patient progress [13]. Digital health care practice, treatment supported by computers or mobile phone apps, and addressing self-monitoring for BPD have been successfully tried on a smaller scale on an ad-hoc basis [14] as an adjunct to trauma work [15]. *Priovi*, a computer program adjunct to Schema therapy for BPD, has recently showed a significant effect on BPD symptoms [16]. Internet-delivered DBT skills training for suicidal and heavy episodic drinkers have also shown feasibility and promise in a pilot randomized control trial (RCT) [17]. In total, 2 different apps targeting DBT skills training have been developed by researchers from University of Washington and Rutgers University, the *DBT coach* [18] and *Pocket skills* [19]. These apps showed promise and acceptability among users. The end users in the *Pocket skills* study voiced a preference for visualization of diary card scores and aggregated scores. The mDiary study seeks to fill this gap in research. The platform and app for the study was developed by the first author and Monsenso. Monsenso has previously developed an app solution aimed at self-monitoring symptoms in the treatment of patients with bipolar disorder [20-22] and has now developed a modified Monsenso platform called the mDiary app.

The objective of this study was to examine the feasibility of using the mDiary app as an adjunct to DBT in the treatment of BPD.

## Methods

### Study Design

Using a mixed-methods approach, the feasibility of the mDiary app was assessed with qualitative interviews in 9 focus groups, as well as evaluations through a questionnaire measuring perceived usability. A total of 5 focus groups were dedicated to therapists only, whereas 4 were dedicated to patients only. Interviews were conducted on-site. Data from the interviews were recorded on an MP3 recorder during the interviews and transcribed verbatim afterwards.

Patients and therapists participating in the focus group interviews were concurrently given the System Usability Scale (SUS) [23] to evaluate system usability. SUS is widely used and is a valid and reliable assessment tool for usability of digital interventions. The total SUS scores are ranked from 0 to 100, where 100 represents the highest usability and a score of 68 reflects an average level of usability [24].

The quantitative data were used to formulate a theory of barriers and facilitators. The grounded theory (GT) approaches [25] of open, axial, and selective coding as well as theoretical sampling, ongoing development, and internally relating of concepts were used in the analysis.

The trustworthiness of the findings, in the sense that a credible and true picture of the phenomenon under scrutiny was presented [26], was addressed by basing conclusions on the verbatim transcripts of the focus groups [26], by discussing the derived concepts among the participating researchers, and by applying a form of triangulation of data by interviewing both therapists and patients and doing this separately, as well as using the SUS scores in the initial formation of a theory of barriers and facilitators. Trustworthiness was increased further by making comparisons of the findings with other broader theories of implementation of technology in the field.

### Participants

All participating patients were enrolled in active DBT treatment in Danish public outpatient psychiatric care from January 2016 to December 2016. Before entering the study, all patients were assessed by a psychiatrist with the International statistical classification of diseases and related health problems. - 10th revision, Fifth edition, 2016 diagnostic manual.

. Patients were eligible for inclusion if they met the criteria for emotionally unstable personality disorder (F60.3), were admitted for psychiatric outpatient treatment, had at least 1 suicide attempt or at least 1 episode of self-harm within the last year, and active problems with suicidal and self-harm urges. Patients were excluded if they had no access to or ability to use a mobile phone or had a comorbid disorder, such as substance abuse, bipolar disorder, or a schizophrenia spectrum disorder. The majority of patients had comorbid disorders, such as depression, anxiety, posttraumatic stress disorder, substance abuse, and obsessive-compulsive Disorder. Alcohol and substance abuse were allowed if it was not the primary diagnosis. After admittance to DBT treatment, BPD symptoms and diagnoses were rechecked again in a separate individual session with an experienced therapist.

The patients’ mean age was 28.0 years (SD 6.2). Half of the sample had completed primary school or less and the other half had secondary education or more. All patients were enrolled in a 12-month DBT program [27] but were at different stages in their treatment program: 10 patients were in the beginning (months 1-3) of their treatment, whereas 6 patients had attended treatment between 4 and 12 months. All study subjects had previously tried paper registration and had switched to app registration for at least 4 weeks.

A total of 23 DBT therapists participated in the study. Half of the therapists were psychiatrists or psychologists and the other half were nurses, occupational therapists, or psychotherapists.

They had DBT therapy experience in the range of 1 to 14 years (mean 6.8 years). Their mean age was 44 years (SD 12). The therapists had different levels of experience with using the mDiary app solution, ranging from 1 month to 1 year.

### Description of the Platform

The mDiary app was customized for dialectical behavior therapy treating borderline personality disorder. This platform consists of 2 modes of data handling: mobile phone-based and Web-based. Patient data were entered by the patient on a mobile app.

#### The Mobile Phone Sections

The mobile phone part of the system retrieves registered data, produces visualizations of that data, and delivers pre-entered psycho-educative material. This app replaces the previously used DBT paper-based diary card. It delivers descriptions of DBT skills in a short text format and in a 3-min sound clip format. Main variables collected by the app are prioritized as mandatory. Mandatory variables can only be modified by Monsenso. In our study, they were day score, dysregulation duration, dysregulation level, emotional numbness, and skill-use. The mandatory ratings were on a 0 to 5 scale. Skills was rated as *learning* or *learned* and could be switched on independently as they were learned. Other variables were optional, for instance: self-harm, drug use, specific basic emotions, and many more. These optional variables could be selected from a long list of typical BPD problem behaviors preprogrammed on the app. Finally, personal customizable variables could be constructed by the user.

**Figure 1:**
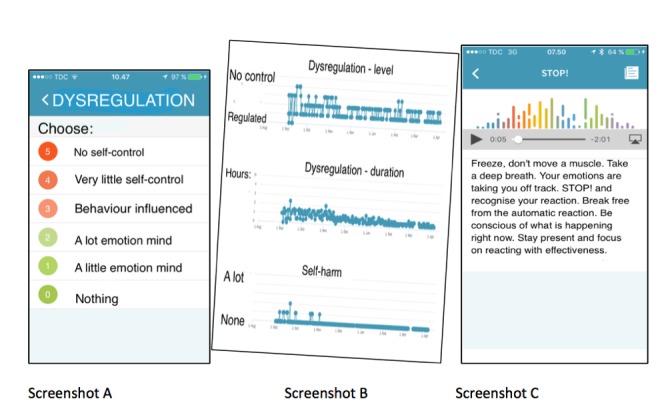
Mobile app screenshots.

Figure 1 shows mobile app screens. Screenshot A is of the daily input screen registering dysregulation level. Screenshot B is an example of a screen visualized after entering the data long term. Screenshot C is a screenshot of the text section and sound clip control buttons for generalizing the skill named *Stop*.

Many other subscreens were available on the mobile phone: daily notes, dedicated questionnaires, medication, triggered notifications, and a large library of psychoeducative material regarding the diagnosis.

#### The Web-Based Overview Screen

The Web-based section of the system has an overview screen intended for a tablet or desktop. This is primarily used in the therapy session to monitor the present state and progress of the patient in treatment. Here, the patient and therapist can explore the entered data together. The same screen can be accessed by patients from home via their computer. The Web-based interface gives a far more detailed current and long-term overview of the treatment progress than what is possible on a mobile phone screen. Submenus of the Web solution part include the following: a time series format overview, diary text overview, and skill utilization overview. For therapists, there is an extra protected section for creation and administration of new patients in the system and the ability to access the data of all the patients the therapist is treating.

**Figure 2:**
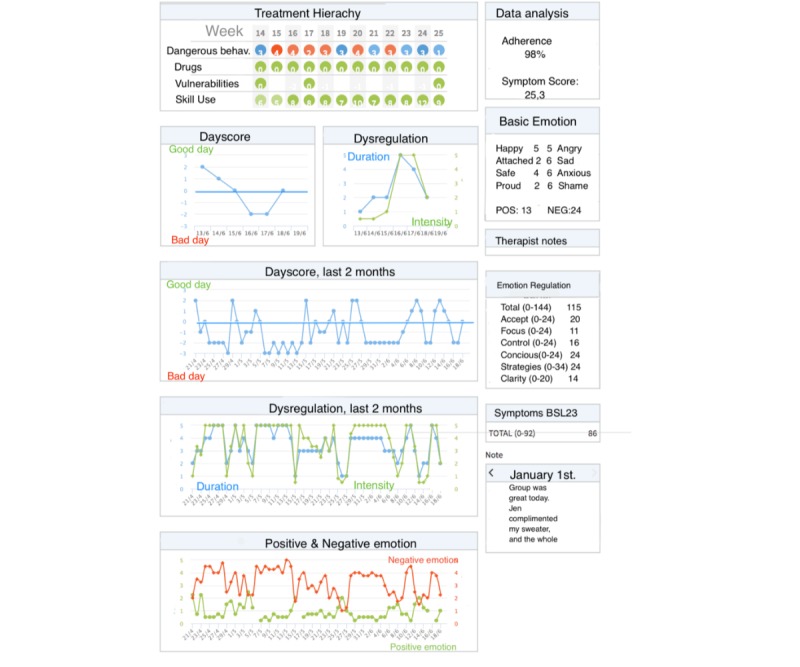
The therapist overview screen.

Figure 2 provides an example of the main therapist overview screen. Here is an example of ratings from a patient who has started previous week out with good days, but then has had higher levels of dysregulated emotion later in the week. It is possible to follow self-harm, drug use, and skill use for the past 12 weeks in the top of the screen. If suicidality or self-harm is present, a red dot marks that week. It can be seen that this patient has had an episode of self-harm or a suicide attempt in week 22, a month ago. At the bottom, total scores of positive and negative affects are displayed, also showing the past 2 months. The right side of the figure shows adherence to registration, symptom scores, results of questionnaire registrations collected by the app, and below that diary text for single days can be seen.

#### Procedure—Development of the App

The primary development site, Site 1, was contacted by the Danish electronic health company, Monsenso, to assist in developing a BPD-specific Web- and mobile phone–based monitoring system. They started developing and testing the basis of the modifications of the already developed platform [21]. As this platform was originally developed for bipolar disorder, adjustments and modifications had to be made. After 4 months, Site 2 was invited to consolidate the improvements made, by testing further in the clinic; 10 months later, 3 new sites were invited to try out the solution in the clinic and report problems back to the app development team.

### Analysis of the Focus Group Interviews

The study followed a GT approach [28]. The duration of all focus group interviews was 1 hour. During the interviews, a whiteboard was divided into a matrix of 2 rows by 3 columns: 2 horizontal rows held *pros and cons*; 3 vertical columns held the themes *paper*, *app*, and *future scenarios*. The participants were given sticky notes and encouraged to place them on the whiteboard and discuss the reason(s) for placing the input as either a *pro* or a *con*. The discussion was moderated by the principal investigator (SHJ) and 1 of the cowriters (TS). Themes from earlier interviews were offered to the participants when statements differed from what was discussed in the focus group (theoretical sampling). The qualitative analysis of the focus groups was performed in different steps as discussed further.

#### In Group

The first part of the analysis was done in collaboration with the patients using sticky notes at the end of the focus group session. The sticky notes that related to each other were grouped into themes by the participants on a whiteboard. This can be seen as the first part of the open coding in the analysis, the identification, and labelling of discrete happenings.

#### After Group

**Figure 3:**
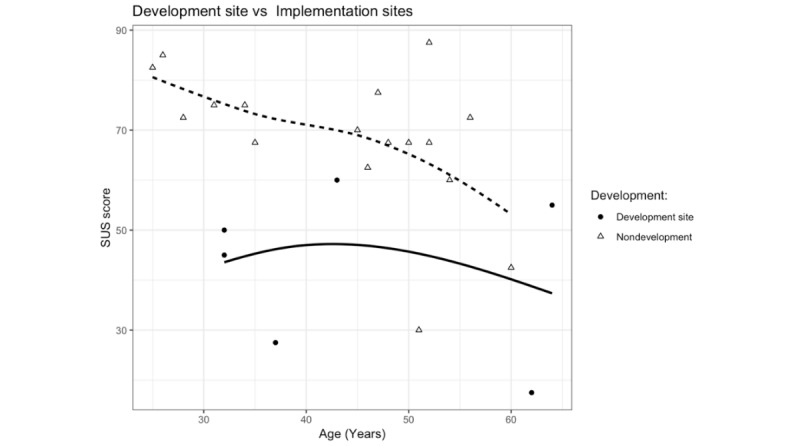
Perceived usability (SUS) in development sites versus age.

**Figure 4:**
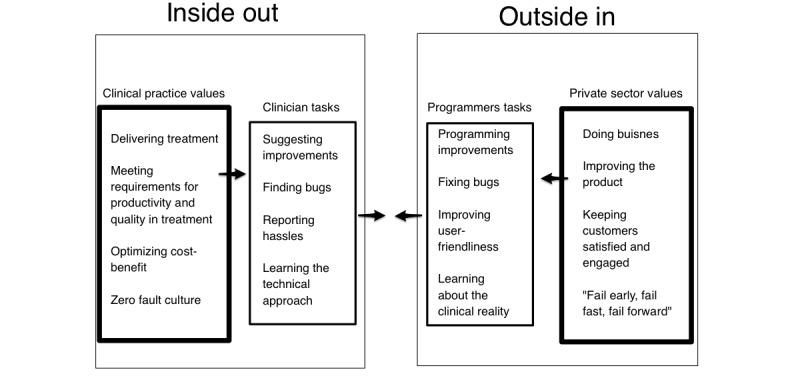
Inside-out and outside-in innovation.

**Figure 5:**
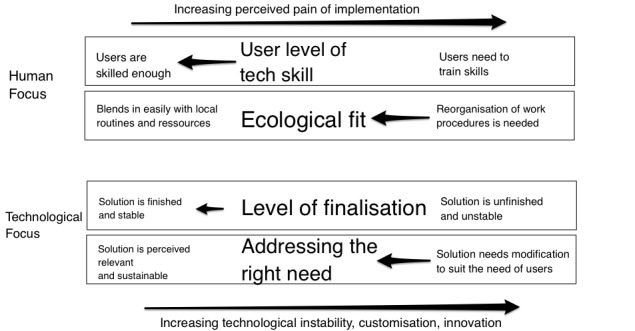
Balancing Inside-out hassles.

The emerging themes and other input were collected for later analysis by the researchers. Questions arising from previous interviews were discussed with the next interview group. After data collection, open coding themes were compared among the different focus groups and after another round of open coding done by the researchers, the resulting categories were arranged into axial coding categories, making connections between the open code labels. These categories were condensed from broader concepts and eventually a core category that we settled on calling *inside-out innovation* (see Barriers and Facilitators in the Results section), arose from the data through selective coding [25]. Perceived usability (SUS) in development sites versus age can be seen in Figure 3. The results of relating the concepts to each other can be seen in Figures 4 and 5.

The research sites were 5 specialized BPD treatment units (Table 1). They were all Danish public outpatient psychiatric facilities treating BPD with DBT.

**Table 1:** Descriptions of participating dialectical behavior therapy sites.

Site number	Role	Duration of app use	Iterative feedback cycles^a^	Number of therapists	Number of patients
1	Primary development site	14 months	70	6	4
2	First testing and feedback	10 months	18	6	5
3	Recently involved	5 weeks	1	4	3
4	Recently involved	1 month	1	3	4
5	Recently involved	1 month	2	4	0

^a^Number of times a suggestion for improvement or a bug was acted upon by adjusting software.

### Ethics

Patients signed an informed consent form before study participation. The study was approved by the Danish Ethics Committee in Region of Southern Denmark (S-20160085). The study protocol was approved by the Danish Data protection Agency (2008-58-0035). The approved database system used was Odense Patient Data Exploratory Network (OPEN) [29].

## Results

### Accepting New Technology

The SUS ratings showed that overall, patients were very satisfied with the solution (Table 2). Therapist SUS ratings were generally significantly lower compared with those of the patients (*P*<.001). Older users tended to rate lower usability than younger users (Pearson correlation coefficient: −0.60). At Site 1, the primary development site, therapists were significantly less satisfied than at the later development sites (*P*=.01), see Table 2 and Figure 3.

As shown in Table 2 and Figure 3, the first development site reported significantly lower usability than the rest of the sites, with scores falling below an SUS score of 60.

The therapists with the most influence on the development were the most critical of the solution. This is probably because they spent the most time dealing with bugs and problems. Our therapist SUS scores suggest that usability improved with every iteration of the development cycle, as more recent adopters had higher SUS scores and were generally more positive in the interviews than early adopters.

**Table 2:** System usability scores for subgroups of users.

Subgroup of users		n	Score, mean (SD)	*P* value^a^
**Subgroup 1**				**<.001**
	Patients	16	81.2 (9.9)	
	Therapists	23	61.6 (18.6)	
**Subgroup 2**				**.01**
	Therapists (development sites)	6	42.5 (16.5)	
	Therapists (nondevelopment sites)	17	68.4 (14.4)	

^a^*t* test, *nonpaired.*

### Barriers and Facilitators

Implementation seen from the outside or from the inside is a matter of perspective. We define *inside-out innovation* as the instability introduced into the treatment context owing to introductions of new work tasks accompanying development and implementation. For instance: learning to use the app, adaptations needed in the context, getting the solution to work technically, and fitting the solution to the needs of the most important work tasks.

This can be seen as a complementary process to the *outside-in innovation*, which means new technology brought to the hospital from an external source. In the mDiary study, the outside-in innovation was the technical solution delivered from Monsenso. They delivered a starting point, a solution that had a general applicability, but were influenced by an outsider’s perspective regarding the specific mental health context it was implemented in.

A central barrier in the development process was found to be the inside-out innovation part.

A suggestion for reconfiguration would typically come from the involved users’ perspective. Figure 4 shows what is seen as valuable from either an outside-in perspective or an inside-out perspective. The smaller squares in the figure show the concrete behavioral actions associated with the 2 perspectives.

When comparing in-clinic innovation with simply implementing a solution already developed and finalized, the effort needed is very different: Reporting hassles and giving suggestions for improvement back to the app developers, upgrading to new versions, reporting technically succinct accounts of bugs back to the app developers (eg, “The bug was on which version of the operating system? What type of phones had the problem? Under precisely what conditions?”), and maybe the clinician encounters trouble accessing data or meet unexpected needs for technological upgrades. All this is time consuming and requires focus and energy, leading to multiple small reductions in time for other tasks. Even if the mDiary study was a time-limited endeavor, this led to frustrations among the clinicians involved in the development. The same demands of productivity during the development phase was expected as within normal operation of the clinic. Mental health workers view delivery of psychosocial treatment as their primary goal. Without clear alignment of expectations and extra resource allocation, this generated frustrations:

I feel that I’m letting the project down. I do not have time to do it properly. I need more training and we have lots of other more important tasks to do, too. I’m not so fast with a smartphone and...It’s a bit embarrassing, and I feel that I come out short, but the time is not there. It has been quite a burden.

Nurse, site 1

In the end, this came down to differences in values: In Figure 4, different views on finding faults in the mDiary app can be traced. The app developers’ perspective finds exploration of faults in the system as a very worthwhile endeavor in accordance with the AGILE project management approach that finds value in *fail early, fail fast, fail forward* [30], whereas therapists typically adhere to a value of minimizing faults at any cost owing to increased patient risk factors [31]. From one perspective, value might mean ability to fail often and fast thereby creating stability and profitability on the long term, whereas from another point of view, value might mean treating a specific patient, here and now, effectively without the delay of reporting bugs back.

### Balancing Acceptance and Change During Inside-Out Innovation

The concept of *balancing acceptance and change* is borrowed from individual DBT psychotherapy [27]. We found this concept helpful to describe barriers and facilitators in the inside-out process. It seems that finding a balance between the status quo and changing old routines is essential and a culprit of many frustrations encountered during implementation.

When improvements were made, new procedures needed to be invented in the clinic, and uncertainty was added to known procedures. It was found that the introduction and development of new technology was a balancing act between *acceptance* and *change*. It could in other words be described as a search for the *optimal level of frustration of the users*. The central task was generating the largest amount of long-term positive change possible while keeping time use optimally focused on delivering therapy to the patients here and now. We have condensed the main acceptance-change dilemmas into 4 main sources of hassle, as shown in Figure 5.

The first 2 dilemmas were related to human factors.

*User level of technical skills*: The first dilemma was out-of-the-box intuitive usability versus training needed before the app was usable to the users. Note that here the term *user* relates to both staff and patients:

I’m not very good at computer stuff, I’m a slow learner.

Therapist

*Ecological fit*: The second dilemma was high versus low need for reorganizing known procedures and daily habits. The slight modification of therapy rules and procedures demanded new procedures at the organizational level:

What happens when the project stops...how can I access the data in 2 years if the patient is admitted again?...will we still have the app?

Therapist

This could imply new difficult-to-solve problems involving both developers and other layers of the hospital administration.

Another aspect of ecological fit could be seen from a patient perspective:

The smartphone is much easier than paper. Most of us bring it along all the time, it’s always there!

Patient

The final two dilemmas were related to technological issues.

*Level of finalization*: The third dilemma was the paradox between having a stable technological product, that is, where the coding is consolidated, versus a more flexible solution where coding is a *work in progress*. Users want both specific tailored solutions suited to the context and at the same time they want the system to be stable. During the development of a new system, it is difficult to have both:

The app is really helpful, but I get really annoyed when things disappear. In the beginning something went wrong, my first 2 weeks of registrations were just lost.

Patient

*Addressing the right need?*: To what extent did the technological solution come across as relevant and sustainable here and now? To what extent does it need modification to effectively address the specific mental health problem the clinician is supposed to solve?:

Why does it need to track my phone calls and my emails? What is it for? I think it’s creepy.

Patient

Monitoring activity by GPS coordinates and counting time spent on talking on the phone were quite easy to do in the solution. Sensors were present in the mobile phones and the coding was already in place as it was used in the bipolar solution. It was not possible to switch this off, and since it was collected passively, it seemed like it did not take extra effort from the patients. The specific task of monitoring mood and skill use, however, did not require this, as it was not a therapeutic necessity. The BPD patients saw this kind of activity monitoring as an unnecessary invasion of their privacy, so even if it was easily accessible and possibly interesting—it was not enough a part of the central task at hand, and thus did not have enough direct relevance to patients. Monsenso, on the other hand, was quite reluctant to let go of this feature. Here, different needs seen from the outside-in and inside-out perspectives stood out clearly. The inside-out perspective seemed to favor utility here and now. The outside-in perspective tended to favor the long-term potential creating big data from the same variables across different diagnosis.

## Discussion

### Principal Findings

The acceptance and usability of the mDiary app was generally high among patients. The SUS scores showed sufficient acceptability among most of the test site therapists. But the primary development site had significantly lower acceptance of the solution. The therapists most involved in the development process were surprisingly the most critical. We have attempted to explain this with a hypothetical concept of inside-out innovation. We found that the most important dilemmas within inside-out implementation were related to user levels of technology skills, ecological fit, finalization of the app, and addressing the right need.

### Accepting New Technology

Resistance to implementing new technology in the health care setting is a well-known problem [32]. Since Davis’ seminal paper in 1989, the Technology Acceptance Models has focused on *perceived usability* and *ease of use* as central variables for the successful implementation of new technology [33,34]. This theory targets implementability by reducing complexity to the individual adopter’s viewpoint. The model has been replicated many times and specific data on health care found ease of use being less important than usability in health care settings [34-36]. Realizing that a more multifaceted approach was needed, Venkatesh et al [37] tested several variables, such as gender, age, experience with performance expectancy, and effort expectancy that could impact the user’s acceptability and satisfaction with technology. They also included the influence of attitude and social factors on behavioral intention to explain resistance more broadly using both background variables of users as well as attitudes. This extended model led to the formulation of the *Unified theory of acceptance and utilization of technology* [37]. In mDiary, usability scores followed this logic as it was found that user age influenced the SUS scores.

### Inside-Out and Outside-In Innovation

Van Gemert-Pijnen et al [38] have pointed out that innovation in mental health is a “collaboratory participatory process of constantly changing cycles.” *Values* in this cooperation are considered central. Values refer to what is considered meaningful in the context, not only economic value. Van Gemert-Pijnen described innovative change using 4 axes: *business model*, *value drivers*, *user requirements*, and *prototyping*. Value specification is considered important in obtaining desirable cooperation: *Value specification implies the recognition and quantification of the economic, medical, social, or behavioral values of the key stakeholders* [38]. Van Gemert-Pijnen’s approach can be thought of as a broader theory looking at implementation from a systems perspective, focusing on different or shared values in the systems’ interaction. Value specification was found to be important in our data. This is most clearly seen in the *addressing the right need* part of Figure 5 and the value sections in Figure 4.

### Balancing Inside-Out Hassles

The hassles captured in the concepts *inside-out innovation* and *ecological fit* have been described in a study by Heeks when he was exploring what he called the design-reality gap [32]. Perspectives differ whether you are a programmer or a user. This is an analog to the *user level of technological skills* dilemma found in Figure 5. The gap in *technology* between design and reality in Heeks’ theory can also be thought of as a case of poor *ecological fit* from our model.

Greenhalgh et al [39] have formulated a theory of “Nonadaptation, abandonment, scale-up, spread, and sustainability of new technology (NASSS).” The values described in Figure 4 fit very well as a theoretical clarification of Greenhalgh’s description of different types of *value propositions*. One value is described by her as “knowledge needed to use the technology.” She suggests that it is helpful to range this from simple to complex needs for knowledge. This is a similar concept to our *user level of technological skills* described in Figure 5. A concept similar to *level of finalization* in the same figure is also addressed by Greenhalgh, who found *dependability* to be a key value [39]. The dilemma of *addressing the* *right need* is covered in her theory as differences in *value propositions*.

This phenomenon of differences in inside-in and outside-in perspectives has also been addressed by Van Gemert-Pijnen et al [38], who also view explication of values of stakeholders as central to successful implementation. The Figure 5 elements of what we call *ecological fit* and *skills needed* are here conceptualized as differences in specification of what has value to whom. The following quote from Van Gemert-Pijnen (p. 10) sums up what we have encountered in developing and testing the mDiary app:

Implementation is often seen as a post design activity. In our view, the conditions for implementation must be considered right from the start (contextual inquiry and value specification). Potential implementation issues, such as limited resources (eg, time, staff, and money) or personal drawbacks (eg, skills, motivation, and anxieties), should be identified. These issues should also be accounted for in the subsequent stages (design and operationalization). In this way, the well-known pitfalls of stakeholder disregard can be avoided [38]

This quote illustrates that the needs related to implementation depend on which perspective you take. The value of implementation is to some extent negotiable and different depending on which stakeholder perspective you take. Designing, implementation, and enhancing usability are all part of the same circular process. In the collaboration between Monsenso and public psychiatry wards, the needs of the patients, the needs of Monsenso, and the needs of therapists were all part of a continuous negotiation: a negotiation of whether to use the severely burdened patients’ time on the long-term implementation of an—as yet untested—system; a negotiation of company resource utilization when improving the technical side of an app with an—as yet unknown ability to generate provenue; and finally, a negotiation with the therapists in getting them to allow for new procedures instead of established procedures of standard DBT treatment. The innovation in the mDiary app was this balancing act, eventually creating an app to the mutual benefit of all involved parties.

### Limitations and Strengths

The study should be interpreted with the following limitations in mind. The number of participants and focus groups was small albeit adequate for a feasibility study. The sample was not randomly selected, but a select sample where patients were given a choice whether they wanted to test the mDiary app as a potential replacement for using paper and pen. Owing to the research method and sample size, the results can only be considered hypothesis-generating. In terms of trustworthiness, it must be noted that group polarization and mutual avoidance of discomfort could influence the results. The patient group is known to avoid difficult emotions [27] as well as being unstable in their baseline emotions [40], which might influence answers toward a more emotional direction. A large part of the sample of therapists were older and very well consolidated in the DBT procedures, which could lead to negative bias regarding approaches aimed at changing well-known procedures [41]. The study also has several strengths, including the involvement of the end users (both patients and therapists) in the development of the platform/app and a thorough and iterative process to optimize the platform/app.

### Conclusions

The mDiary app is a useful and acceptable way of registering DBT diary cards, tracking emotion regulation, and skill acquisition and is now ready for implementation. Our data suggest adequate usability and feasibility in clinical departments with higher perceived usability from patients compared with therapists. At the present stage, the app is sufficiently ready to be used in further studies evaluating effectiveness. This will be done in the mDiary RCT study.

## Abbreviations

BPD: borderline personality disorder
DBT: dialectical behavior therapy
GT: grounded theory
RCT: randomized control trial
SUS: System Usability Scale

## References

[1] Høye A, Jacobsen BK, Hansen V (2013). Sex differences in mortality of admitted patients with personality disorders in North Norway—a prospective register study. BMC Psychiatry.

[2] Wahlbeck K, Westman J, Nordentoft M, Gissler M, Laursen TM (2011). Outcomes of Nordic mental health systems: life expectancy of patients with mental disorders. Br J Psychiatry.

[3] Torgersen S, Kringlen E, Cramer V (2001). The prevalence of personality disorders in a community sample. Arch Gen Psychiatry.

[4] Ekselius L, Tillfors M, Furmark T, Fredrikson M (2001). Personality disorders in the general population: DSM-IV and ICD-10 defined prevalence as related to sociodemographic profile. Pers Individ Dif.

[5] Stoffers JM, Völlm BA, Rücker G, Timmer A, Huband N, Lieb K (2012). Psychological therapies for people with borderline personality disorder. Cochrane Database Syst Rev.

[6] Widiger TA (2006). The Oxford Handbook of Personality Disorders.

[7] Binks CA, Fenton M, McCarthy L, Lee T, Adams CE, Duggan C (2006). Psychological therapies for people with borderline personality disorder. Cochrane Database Syst Rev.

[8] Choi-Kain LW, Finch EF, Masland SR, Jenkins JA, Unruh BT (2017). What works in the treatment of borderline personality disorder. Curr Behav Neurosci Rep.

[9] Stiglmayr C, Stecher-Mohr J, Wagner T, Meiβner J, Spretz D, Steffens C, Roepke S, Fydrich T, Salbach-Andrae H, Schulze J, Renneberg B (2014). Effectiveness of dialectic behavioral therapy in routine outpatient care: the Berlin borderline study. Borderline Personal Disord Emot Dysregul.

[10] Rizvi SL, Thomas MC, Friedman HS (2015). Dialectical behavior therapy. Encyclopedia of Mental Health.

[11] Eist H, Linehan MM (2015). DBT Skills Training Manual. Second Edition.

[12] Rizvi SL, Dimeff LA, Skutch J, Carroll D, Linehan MM (2011). A pilot study of the DBT coach: an interactive mobile phone application for individuals with borderline personality disorder and substance use disorder. Behav Ther.

[13] Chan S, Torous J, Hinton L, Yellowlees P (2014). Mobile tele-mental health: increasing applications and a move to hybrid models of care. Healthcare (Basel).

[14] Jahng S, Wood PK, Trull TJ (2008). Analysis of affective instability in ecological momentary assessment: indices using successive difference and group comparison via multilevel modeling. Psychol Methods.

[15] Görg N, Priebe K, Deuschel T, Schüller M, Schriner F, Kleindienst N, Ludäscher P, Schmahl C, Bohus M (2016). Computer-assisted in sensu exposure for posttraumatic stress disorder: development and evaluation. JMIR Ment Health.

[16] Jacob GA, Hauer A, Köhne S, Assmann N, Schaich A, Schweiger U, Fassbinder E (2018). A schema therapy-based ehealth program for patients with borderline personality disorder (priovi): naturalistic single-arm observational study. JMIR Ment Health.

[17] Wilks CR, Lungu A, Ang SY, Matsumiya B, Yin Q, Linehan MM (2018). A randomized controlled trial of an internet delivered dialectical behavior therapy skills training for suicidal and heavy episodic drinkers. J Affect Disord.

[18] Rizvi SL, Linehan MM (2005). The treatment of maladaptive shame in borderline personality disorder: a pilot study of 'opposite action'. Cogn Behav Pract.

[19] Schroeder J, Wilkes C, Rowan K, Toledo A, Paradiso A, Czerwinski M, Mark G, Linehan MM (2018). Pocket Skills: A Conversational Mobile Web App To Support Dialectical Behavioral Therapy. Proceedings of the 2018 CHI Conference on Human Factors in Computing Systems.

[20] Frost M, Doryab A, Faurholt-Jepsen M, Kessing LV, Bardram JE (2013). Supporting Disease Insight Through Data Analysis: Refinements of the Monarca Self-Assessment System. Proceedings of the 2013 ACM International Joint Conference on Pervasive and Ubiquitous Computing.

[21] Faurholt-Jepsen M, Frost M, Vinberg M, Christensen EM, Bardram JE, Kessing LV (2014). Smartphone data as objective measures of bipolar disorder symptoms. Psychiatry Res.

[22] Bardram JE, Frost M, Szántó K, Faurholt-Jepsen M, Vinberg M, Kessing LV (2013). Designing Mobile Health Technology for Bipolar Disorder: A Field Trial of the Monarca System. Proceedings of the SIGCHI Conference on Human Factors in Computing Systems.

[23] Brooke J, Jordan PW, Thomas B, McClelland IL, Weerdmeester B (1996). SUS - a quick and dirty usability scale. Usability Evaluation In Industry.

[24] Lewis JR (2018). The system usability scale: past, present, and future. Int J Hum-Comput Int.

[25] Corbin J, Strauss A (2019). Basics of Qualitative Research: Techniques and Procedures for Developing Grounded Theory. Third Edition.

[26] Shenton AK, Coghlan D, Brydon-Miller M (2014). Strategies for ensuring trustworthiness in qualitative research projects. The Sage Encyclopedia of Action Research.

[27] Linehan MM, Wilks CR (2015). The course and evolution of dialectical behavior therapy. Am J Psychother.

[28] Charmaz K, Henwood K, Willig C, Wendy SR (2017). Grounded theory methods for qualitative psychology. The Sage Handbook of Qualitative Research in Psychology.

[29] Odense Patient Data Exploratory Network: OPEN.

[30] Schwaber K, Sutherland J (2012). Software in 30 Days: How Agile Managers Beat the Odds, Delight Their Customers, and Leave Competitors in the Dust.

[31] Ingerslev K, Bjorn K, Johansen J (2012). Innovating the Capacity to Innovate. Proceedings of the 1st International Conference on Innovation and Entrepreneurship in Health.

[32] Heeks R (2006). Health information systems: failure, success and improvisation. Int J Med Inform.

[33] Davis FD, Bagozzi RP, Warshaw PR (1989). User acceptance of computer technology: a comparison of two theoretical models. Manag Sci.

[34] Ward R (2013). The application of technology acceptance and diffusion of innovation models in healthcare informatics. Health Policy Technol.

[35] Raitoharju R (2007). Information Technology Acceptance in the Finnish Social and Healthcare Sector: Exploring the Effects of Cultural Factors.

[36] Schaper LK, Pervan GP (2007). ICT and OTs: a model of information and communication technology acceptance and utilisation by occupational therapists. Int J Med Inform.

[37] Venkatesh V, Morris MG, Davis GB, Davis FD (2003). User acceptance of information technology: toward a unified view. MIS Q.

[38] van Gemert-Pijnen JE, Nijland N, van Limburg M, Ossebaard HC, Kelders SM, Eysenbach G, Seydel ER (2011). A holistic framework to improve the uptake and impact of ehealth technologies. J Med Internet Res.

[39] Greenhalgh T, Wherton J, Papoutsi C, Lynch J, Hughes G, A'Court C, Hinder S, Fahy N, Procter R, Shaw S (2017). Beyond adoption: a new framework for theorizing and evaluating nonadoption, abandonment, and challenges to the scale-up, spread, and sustainability of health and care technologies. J Med Internet Res.

[40] Santangelo PS, Koenig J, Kockler TD, Eid M, Holtmann J, Koudela-Hamila S, Parzer P, Resch F, Bohus M, Kaess M, Ebner-Priemer UW (2018). Affective instability across the lifespan in borderline personality disorder - a cross-sectional e-diary study. Acta Psychiatr Scand.

[41] Klein KJ, Knight AP (2016). Innovation implementation. Curr Dir Psychol Sci.

